# Building the bridges: molecular mechanisms of tunneling nanotube formation

**DOI:** 10.1007/s00018-026-06097-1

**Published:** 2026-04-24

**Authors:** Christel Brou, Chiara Zurzolo

**Affiliations:** Unité de Trafic Membranaire et Pathogénèse, Département de Biologie Cellulaire et Infection, Institut Pasteur, CNRS UMR3691, Université Paris Cité, Paris, France

**Keywords:** Tunneling nanotubes, Cell-cell communication, Actin regulators, Tetraspanins, GTPases

## Abstract

Tunneling nanotubes (TNTs) are thin, actin-based conduits that provide direct cytoplasmic continuity between cells, enabling the transfer of ions, proteins, organelles, and pathogens. Although discovered nearly two decades ago, the molecular mechanisms that govern TNT biogenesis are only beginning to be resolved. Recent studies have highlighted two main formation modes: filopodia-like protrusion and cell dislodgement. Both likely involve similar molecular steps, but in different sequences. These include regulation by actin dynamics, Rho and Rab GTPases, motor proteins, and membrane-associated adaptors. Environmental stressors such as hypoxia, infection, and inflammation strongly stimulate TNT formation, underscoring their role in adaptation and survival. Understanding the molecular logic of TNT assembly is essential, as these structures influence development, immunity, cancer progression, and neurodegeneration. In this review, we summarize current knowledge of the signaling pathways, cytoskeletal regulators, and membrane remodeling events that drive TNT formation, and discuss outstanding questions.

## Introduction

One way in which cells communicate with their environment is by building temporary and specialized structures that can engulf materials and eventually deliver them over short or long distances. These cell appendages include extracellular vesicles and particles, filopodia, lamellipodia, migrasomes, and tunnelling nanotubes (TNTs). The latter were first described in 2004 by HH Gerdes’s laboratory [1] using cultured PC12 cells. They were defined as fragile nanotubular structures formed de novo between cells that do not contact the substrate and facilitate the selective transfer of membrane vesicles.

Since then, the definition of TNTs has been only slightly refined. They are defined as hollow, labile membrane protrusions that connect cells over distances of between 10 and 100 μm. They hover over the substrate in 2D cell cultures and are supported by actin. Their diameter ranges from 50 to 900 nm, and their open ends allow direct cytoplasmic continuity enabling transfer of various cargo (extensively described in [2]). Therefore, F-actin serves as a structural scaffold and a track for active cargo transport.

TNTs have been observed in many cultured cell types (see [3] for a complete list of cultured cells). To our knowledge, no cultured cell line has been found to be incapable of forming them. TNTs must, however, be distinguished from neurites, filopodia, and cytokinetic bridges formed between dividing cells. Because of these uncertainties, similar structures have been described in various contexts and named differently: long filopodia, intercellular connections, photoreceptor nanotubes for example (summarized in [4]). In some cases, parallel structure and function studies allowed to conclude that TNT-like connections do exist in vivo, both in development and adult tissues, as well as in pathological contexts.

In mammals, TNTs were first visualized in the corneal stroma, whose transparent collagen matrix and lack of pigmentation made these fragile structures detectable [5]. As reviewed by Chinnery and Kellers [6], TNTs have also been identified in the mouse dura mater, another dense connective tissue with structural similarities to the cornea; the trabecular meshwork; retinal pericytes; and the retinal pigment epithelium (RPE) where they might contribute to ocular homeostasis. In disease pathogenesis, their regulation can be affected by mitochondrial dysfunction and acute or chronic inflammation, which occur in a wide variety of ocular diseases. Connections between pairs of granule neurons in the cerebellum of postnatal mice were identified using 3D serial sectioning scanning electron microscopy [7]. Recently, dendrite-dendrite nanotubes (DNTs), which are actin-based and able to propagate calcium ions and proteins such as Amyloid-β, were identified in the mature brains of mice and humans. These structures were first visualized from volumetric electron microscopy datasets and confirmed in mouse dissociated cortical neuron cultures and thin mouse brain slices [8].

During development, mosaic labeling in zebrafish embryos revealed protrusions fulfilling several TNT criteria, including bidirectional transfer of proteins, endosomes, and mitochondria, and responses to known TNT inducers [9]. In mice, TNT-like connections (TNTLs) extend from cardiomyocytes to endocardial cells through the cardiac jelly, enabling EC–CM communication essential for trabecular initiation. Serial block-face SEM confirmed that these structures contain actin bundles and vesicles, consistent with TNTs, and mediate trafficking of cytoplasmic proteins and Notch ligands required for ventricular patterning [10].

TNTs have also been implicated in cancer. In vivo imaging of glioblastoma stem-like cells in mice showed that tumor cells extend ultra-long protrusions to interconnect, proliferate, and invade, conferring resistance to therapy [11, 12]. Organoids derived from glioblastoma stem-like cells revealed networks composed of both tumor microtubes (TMs) and TNTs, with mitochondrial transfer restricted to TNTs [13].

One outstanding question in the field is: what are the functions of TNT-like structures, given that their presence challenges the view of cells as isolated entities? Their roles are not fully understood, but TNT can transfer a wide range of cargo, including mRNAs, microRNAs, organelles, protein aggregates, and viruses (reviewed in [3, 14]). Mitochondrial transfer is particularly significant: it can influence both donor and recipient cells and is often directional [15–17]. In glioblastoma, for example, cancer cells hijack mitochondria from surrounding immune cells such as T cells [18]. Chondrocytes under oxidative stress can acquire mitochondria from mesenchymal stromal cells to restore bioenergetics [19, 20]. In models of neurodegenerative disease, TNTs appear to play a dual role in disease progression and neuroprotection. On the one hand, they facilitate the intercellular propagation of misfolded proteins, thereby contributing to the spread of pathology [8, 14, 21]. On the other hand, they may exert neuroprotective functions by enabling the transfer of healthy mitochondria to stressed neurons, which reduces aggregate burden [22, 23].

How directionality is imposed during TNT formation remains unknown. Moreover, studies that describe the full range of materials transferred in a specific context, as well as their biological impact on donor and recipient cells, remain technically challenging and have yet to be conducted. Most of the current literature on the functionality of TNTs, which is defined as the ability of one cell to donate cellular material to a connected cell, uses one specific type of marker (labelled mitochondria, vesicles or RNA) to monitor transfer, regardless of the actual set of transferred cargoes (if any).

Structurally, TNTs show considerable diversity [4]. While F-actin is a core component, they may also contain microtubules or intermediate filaments, which can be twisted around each other [24–28]. The presence of microtubules and intermediate filaments correlate with greater thickness [15, 29]. Microtubules could allow the transport of larger organelles, such as mitochondria and lysosomes, but this has not been consistently demonstrated and may depend on cell type. Similarly, the half-life of TNTs appears to vary from cell to cell, ranging from minutes for neural cells to several hours for normal rat kidney cells [1] and even to 24 h in alphaherpes virus-induced, microtubule-containing TNTs [26]. Additionally, TNTs are not always open-ended, but it is possible that closed TNT-like structures are either TNTs in the process of formation or completely different cellular structures.

This review summarizes the current knowledge of the molecular mechanisms that govern the formation of TNTs and highlights the outstanding questions about their biology and physiological importance.

## Ultrastructure and composition of TNTs

The first description of TNTs was provided by live confocal microscopy images of cells labelled with wheat germ agglutinin for the plasma membrane, phalloidin for actin, and dye-labelled organelles [1]. The ultrastructure of TNTs was examined using SEM and TEM, which revealed that their membrane appeared to be continuous with the membranes of connected cells; therefore, TNTs are open [1].

These results were further confirmed by numerous studies, which are listed in Cordero and Zurzolo [4]. However, setting up a new protocol to preserve the ultrastructure of TNTs was a step forward in revealing that, by combining Cryo-correlative light and electron microscopy (cryo-CLEM) with cryo-electron microscopy (cryo-TEM) and cryo-electron tomography (cryo-ET), TNTs are comprised of a bundle of several thin individual TNTs (iTNTs) braided together and containing cargoes [30]. The actin polarity inside each iTNT can be opposite, allowing for bidirectional material exchange. Since iTNTs are held together by linkers that are partially composed of N-cadherin, it has been hypothesized that they form by running on top of each other and becoming braided into more rigid bundles. These structures are seen as single TNTs by light microscopy. Notably, actin filaments run uninterrupted for long distances in iTNTs, whereas in the filopodia of the same cells they are much shorter (around 1 μm) [30]. This peculiar ultrastructure of iTNTs suggests that they are regulated completely differently from the beginning of their formation and are not the result of the fusion of a subset of filopodia. The various thicknesses of TNTs can result from their content of filamentous actin (F-actin), microtubules, or intermediate filaments, as well as from their arrangement into bundles of iTNTs. iTNTs have only been described in neuronal murine (CADs) and human SH-SY5Y cells [30], as well as in HS-5 stromal cells [31]. This organization may also depend on the cell type.

TNTs are membrane tunnels supported by the actin cytoskeleton. However, their content is, of course, more complex in terms of the composition of the membrane (lipids and associated proteins) and the materials inside. The lipid composition of TNTs remains largely unexplored, although cholesterol and sphingolipids were enriched in TNT connecting urothelial cells [32]. Only two studies have attempted to describe the full protein composition of TNTs compared to other protrusions or EVs, using completely different approaches [33, 34]. The first study used laser capture microdissection (LCM) to isolate and enrich cellular protrusions from fixed CAD cells of a mouse neuronal origin, before submitting the fractions to mass spectrometry [33, 35]. By applying this method to both undifferentiated cells, which exhibit probable TNTs induced by H₂O₂ treatment, and differentiated cells, which exhibit axonal and dendritic protrusions, the authors were able to identify proteins that belong to TNTs. TNTs were found to have the most unique set of proteins, with 74% of protein hits being exclusive to TNTs compared to other protrusions. The other approach took advantage of the fact that TNTs are sensitive to mechanical stress because they lack attachment to the substrate. A protocol was established to break the TNTs and isolate them from the cell bodies and extracellular vesicles [34]. Mass spectrometry analysis identified over 1,000 proteins in the TNT-enriched fraction (TNTome). Notably, almost all of the few known regulators or components of TNTs were identified by this approach [34]. Interestingly, of the 190 proteins identified by Gousset et al. (2019) in two hCAD samples enriched in TNTs, 101 were also found in the TNTome, mostly corresponding to the most abundant cellular proteins [33, 34]. Together, these two studies demonstrate that TNTs have a specific composition, opening up new possibilities for identifying TNT markers and regulators. However, these experiments did not distinguish between material transferred in TNTs and actual regulators or core components.

## Methods for identifying factors involved in TNT formation and regulation

The TNT field suffers from a lack of specific molecular markers for these structures. Additionally, they are fragile and tend to be destroyed during sample preparation unless specific protocols are employed. This is why identifying them in complex environments (2D neuronal cultures, 3D cell cultures, tissues and tumors) is very difficult and requires several criteria to be met [4]. First, the connections identified under light phase or using a fluorescent membrane marker and confocal microscopy should be positive for actin and should not touch the substrate in the case of 2D cell culture. Negative staining could help distinguish TNTs from similar protrusions. For neurons whose dendrites could be mistaken for TNT, negative labeling for dendritic (MAP-2) and axonal (β-III-tubulin) markers was an additional criterion used by Vargas, Loria, et al. [36] in their study of primary neurons at an early stage. Chang et al. [8] identified dendritic nanotubes as negative for neurofilament H and Tau. To discriminate TNTs from cytonemes and cytokinetic bridges in vivo, Korenkova et al. have used Wnt8 and Cep55 labeling respectively [9]. Secondly, TNTs are characterized by their ability to transfer cellular material, which can be assessed in various ways: in vivo imaging of cytoplasmic transfer or labelled vesicles [34], imaging after fixation, and quantitative measurements using two distinguishable cell populations (one donating material and the other receiving it). The adapted protocols have been thoroughly described in various papers [37–40]. Super-resolution imaging using STORM or more sophisticated lifetime imaging pipelines were employed to reveal the structural and functional characteristics of fixed and living TNTs at the nanometer scale [27, 41]. A summary of all the techniques used in vivo to identify TNTs, as well as the type of connections identified, can be found in Palese et al. [3]. Because fluorescence microscopy has a limited resolution, demonstrating cytoplasmic continuity between connected cells and the presence of iTNTs requires SEM or cryo-EM. Using electron microscopy approaches allowed also to get insight into structural features of TNTs, as summarized in [4]. A morphological characterization was recently performed using supervised deep learning on volumetric, thin brain slice SEM datasets [8].

When it comes to the identification of TNT regulators, most of the ones described in literature have been chosen based on their known involvement in filopodia formation and the regulation of actin polymerization and stability, as well as their ability to bend the membrane. A recent proteomic analysis of TNTs from U2OS cells allowed the identification and characterization of the tetraspanins CD9 and CD81 as major regulators of TNT formation. This analysis also provided a long list of potential TNT regulators that require confirmation [34].

To evaluate a factor’s role in TNT formation or function, researchers conduct classical invalidation or overexpression experiments and measure the resulting effects on the cell connections (see Table 1). Depending on how the number, functionality, or stability of the TNTs is affected, one can draw conclusions about their participation in one of the steps of TNT formation (described in the next section). Similarly, epistatic experiments determine whether factors act in the same pathway and in what order or independently of each other. However, a comprehensive model establishing how the identified factors interact with each other is not yet available. Similarly, no protein has been proven to be essential for TNT formation and function. Most studies show a change in TNT number or activity, but never complete abolition, when the factor of interest is knocked out. This probably reflects the fact that various pathways could be redundant for TNT formation and function.

**Table 1.**
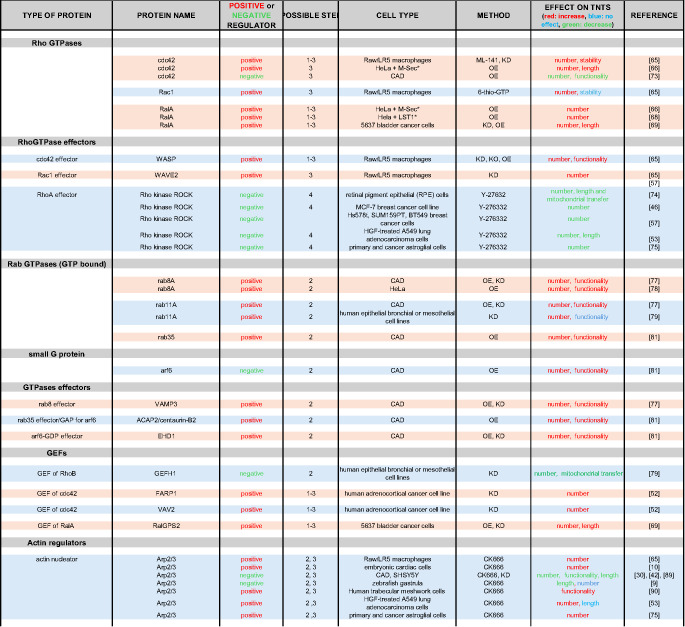

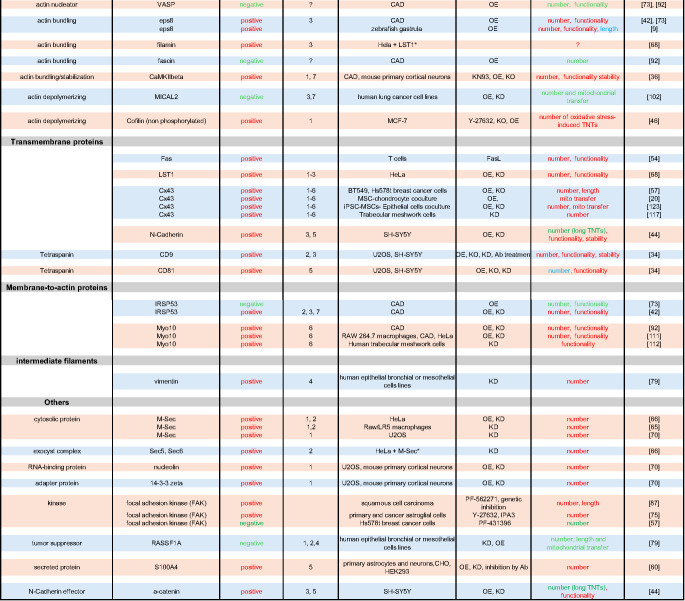
Regulators of TNT formation and their function as identified in vitro. The known regulators (column 2) are organised by protein type (column 1). Their overall action on TNT regulation is indicated in column 3 (positive or negative). The column 'Effects on TNTs' summarises the results of the various ways in which TNTs could be affected, such as their number, length, functionality (i.e. the ability to transfer cellular material) and stability. The methodology used and the cellular model are indicated in columns 5 and 6, respectively, for each factor. In the “Cell type” column, the asterisks indicate that the named cells also express exogenous proteins (e.g. M-Sec and LST1). The numbers in the "Possible Step" column correspond to the steps described in Fig. 1 and the main text, controlled by the protein of interest. OE: overexpression; KD: knock-down; KO: knock-out. ML-141, 6-thio-GTP, Y-27632, CK666, KN93, PF-562271 and PF-431396, IPA3 are inhibitors of cdc42, rac, ROCK, ARP2/3, CaMK, FAK and PAK respectively. HeLa is an epithelial cell that was isolated from the cervix of a woman with adenocarcinoma. CAD (Cath. -a-differentiated) cells are a variant of a CNS catecholaminergic cell line established from a brain tumour in a transgenic mouse carrying wild-type SV40 T antigen under transcriptional control of rat tyrosine hydroxylase promoter. MCF-7 (Michigan Cancer Foundation-7) is a hormone-dependent, and estrogen and progesterone receptor positive breast cancer cell line. SH-SY5Y cells are a cloned subline of a human neuroblastoma cell line that can be differentiated from a neuroblast-like state into mature human neurons. U2OS is a cell line with epithelial morphology that was derived from a moderately differentiated human sarcoma. Human Embryonic Kidney (HEK) 293 is a cell line that was isolated from the kidney of a human embryo. Chinese hamster ovary (CHO) cells are defined as cell lines derived from the ovarian cells of the Chinese hamster

## Steps underlying TNT formation

**Fig. 1:**
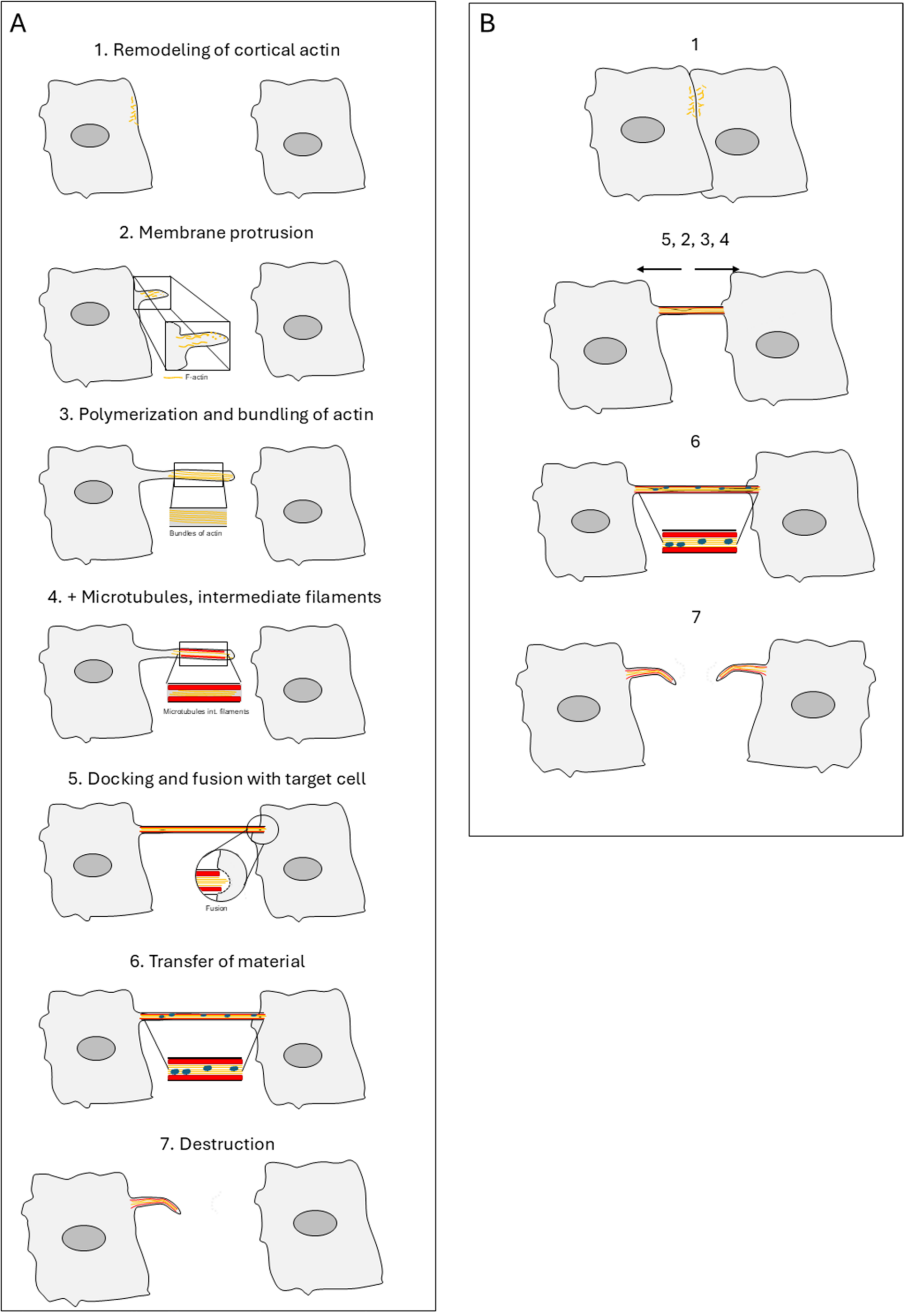
The steps involved in TNT formation are shown in (**A**) according to the protrusion-forming mode and in (**B**) by cell dislodgement. Steps 1 to 7 are described in A and in the main text. In B, the numbers correspond to the same steps, but the order in which they are arranged is not well established

TNT formation depends on dynamic remodeling of the actin cytoskeleton and plasma membrane, driven by signaling cues and stress. Traditionally, two modes of formation have been described [1]. The first is de novo filopodia-like extension, whereby one cell extends a protrusion towards a target cell (Fig. 1). After contact is made, the protrusion fuses with the recipient cell’s membrane to establish a continuous nanotube. The second mode is cell distanglement, whereby two cells that were previously in contact begin to migrate apart, leaving behind intercellular membrane bridges that evolve into TNTs. These two types of mechanism have been observed in different cell types, CAD cells [42], PC12 [1, 43] for the first, and SH-SY5Y [44] cells for the second. However, it is unclear whether each cell type uses a specific mechanism or if this depends on other parameters, such as cell density, the inducing signal or imaging parameters. In vivo in zebrafish gastrula, both mechanisms were observed [9], demonstrating the high plasticity of TNT formation mechanisms.

Nevertheless, common events necessary for building a TNT can be identified, with the order of their occurrence varying slightly depending on the model (see Fig. 1).

1. Breaking and remodeling of cortical actin, necessary to create the space in which the orthogonal structure can grow.
2. Formation of a membrane protrusion, possible if membrane lipids are provided and membrane is bent at the same time actin polymerizes.
3. Polymerization of actin, mainly without branching, creating and elongating a bundle of actin filament that will push the membrane and grow the protrusion.
4. Possibly, addition of other cytoskeleton components (microtubules and intermediate filaments).
5. Tethering, docking, and fusion with the target cell.
6. Transfer of material using specific motors.
7. Destruction of TNTs, which could also be regulated given that their stability varies between cell types.

In the following chapters, we will summarize what is known about the actors involved in TNT growth and attempt to understand which steps they could induce or regulate. It should be acknowledged that it is very difficult to draw a unified mechanistic model, since the current results were obtained using different cell types, techniques, and even definitions of TNTs. Additionally, various pathways and redundant actors are probably involved in the formation of TNTs. The results are summarised in Table 1, which shows for each factor the cell types used, the method employed to specifically target the protein of interest and the resulting effect on TNT biology (e.g. number, physical characteristics, functionality and stability), as well as the possible regulated step in TNT formation. The table illustrates that some factors have been reported to affect TNTs positively or negatively, depending on the assays and cellular model used.

## Induction of TNT growth

TNTs have been shown to be induced by stress signals in vitro. These signals include hypoxia [17, 45] and oxidative stress, induced by drugs such as doxorubicin [46], staurosporine or H₂O₂ [28], but also nutrient deprivation [17], chemical exposure, ionizing radiation or temozolomide [47], inflammation, protein aggregates [14], and virus infection (e.g., HIV in [48]; SARS-CoV-2 in [49]; Pseudorabies Virus US3 in [25]; Influenza A virus in [50]). Some signaling pathways have also been shown to affect TNT formation and function. Wnt signaling [36, 51] stimulates TNTs in neuronal cells independently of the β-catenin effector but through CaMKII [36]; Gi/o-coupled GPCRs [52] are affected by their natural agonist, 5-oxo-eicosatetraenoic acid (5-oxo-ETE), in a human adrenocortical cancer cell line through the transactivation of the epidermal growth factor receptor (EGFR) and elevated intracellular Ca²⁺ levels. Other pathways include HGF-Met/B1 integrin in A549 cells [53] and Fas stimulation of T lymphocytes [54].

Cellular stresses have various consequences beyond the TNT response. For example, they can lead to the release of extracellular particles, such as exosomes [55], which, like TNTs, participate in long distance communication between cells [56]. Exosomes released from breast cancer cells, in turn, promote TNT biogenesis [57, 58]. Conversely, membrane-bound exosomes that attach to the plasma membrane of brain endothelial cells (BECs) participate in TNT formation between cells by either inducing TNT formation or fusing together [59]. The interplay between exosomes and TNTs, which may lead to a synergistic effect on intercellular communication, is not yet fully understood.

The mechanisms and molecules involved in how specific cells are connected through TNTs are still poorly understood. This could be based on the health or content imbalance of the two connected cells. However, how TNTs or cells can sense these differences and the possible beneficial effects of connecting to a specific target cell is still unclear. Only one chemoattracting molecule was described in 2012 [60]. The calcium-binding protein S100A4, which is secreted, was shown to induce directional guidance of TNTs between astrocytes and neurons and in cells that overexpress it. It acts through its receptor, RAGE (receptor for advanced glycation end products). The gradient of S100A4 would be created by the cleavage of S100A4 produced in the TNT-initiating cell due to caspase-3 activation. Meanwhile, the target cells have a relatively high concentration of S100A4 around them [60]. One could hypothesize that molecules that work like S100A4 would affect the tethering of the growing TNT to the cellular membrane (step 5).

## Signaling molecules

### Small Rho GTPases

The signaling pathways shown to be involved in TNT induction (see above) culminate in the activation of Rho-small GTPases, leading to the assembly and disassembly of the actin cytoskeleton. Typical small G proteins include 4 main members, RhoA, RhoG, cdc42 and Rac1. These proteins drive signal platforms for the formation of stress fibers, podosomes, filopodia and lamellipodia respectively [61, 62]. These proteins can switch from an active state (GTP-bound) to an inactive state (GDP-bound) under the control of guanine nucleotide exchange factors (GEFs), of GTPase activating proteins (GAPs) and of guanine nucleotide dissociation inhibitors (GDIs). GEFs favor the exchange of GDP for GTP, activating the Rho GTPases, inversely GAPs inactivate them. GDIs extract GTPases from membranes, sequester them in an inactivated state and protect them from degradation. Therefore, Rho GTPases reversibly shuttle between the plasma membrane where they are active and the cytosol where they are inert. The switch between the GDI-bound and free states of Rho GTPases regulates their arrival rate at membranes, where they can accumulate and be activated by GEFs [63]. Their activation leads to the activation and clustering of membrane-bending proteins, as well as the local recruitment of actin polymerizers and bundlers. This process ultimately enables the growth of a protrusion. The large number of GEFs and GAPs (145) and their potential combinations contribute to the dynamic control of cytoskeletal outcomes. Due to the specific spatial distribution of GEFs and GAPs, Rho signaling responses in cells are highly localized, spatially confined and have been observed in distinct subcellular zones. Additionally, several GTPases can cross talk and operate simultaneously, and multi-protein complexes can form between GEFs and GAPs. As demonstrated for Rac1 signaling from integrin adhesions [64], various GEFs/GAPs could be activated at different stages/locations during TNT formation. This leads to the spatiotemporal regulation of actin regulators and the regulation of different TNT formation stages. The same study identified 34 actin-associated RhoGEFs and RhoGAPs, among which cdc42 regulators were overrepresented. Accordingly, published data show that CDC42 has primarily been described as a regulator of TNTs, though the data are contradictory.

Hanna et al. used multiple imaging techniques, including super-resolution microscopy (3D-SIM) and live-cell imaging with FRET-based Rho GTPase biosensors to visualize TNT formation in Raw/LR5 macrophages [65]. These techniques allowed them to detect the spatiotemporal dynamics of Rho GTPases with high resolution within live single cells. They found that the formation of TNTs required the activity and differential localization of Cdc42 (located at the base of the TNTs) and Rac1 (localized throughout the TNT). Downstream Rho GTPase effectors, such as the Wiskott-Aldrich syndrome protein (WASP) and the WASP family verprolin-homologous protein 2 (WAVE2), are also important, and these pathways act together during TNT biogenesis. Cdc42 may be necessary for the initial formation and persistence of TNTs, possibly by activating its downstream effector, WASP. Rac1 activity, on the other hand, may be necessary for TNT maintenance [65].

M-Sec, also known as TNFaip2 (tumor necrosis factor-α-induced protein 2) or B94, was tested as a TNT regulator because it is highly expressed in the mammalian myeloid lineages, including dendritic cells and macrophages, and is upregulated by inflammatory stimuli, such as lipopolysaccharide or interferon-γ, which are conditions that are known to enhance TNT formation [66]. Interestingly, when overexpressed in HeLa cells, M-Sec—present in the cytoplasm and inside TNTs—promoted TNT growth by binding to and signaling through active RalA and, to a lesser extent, CDC42, but not Rac1. Since cdc42 inhibition affected the length of TNTs, it was proposed that cdc42 would be necessary for elongation. Furthermore, knocking down Sec5 or Sec6, two components of the exocyst complex, significantly impaired M-Sec-induced TNT formation. This was associated with a reduction in calcium flux propagation. The Exocyst complex, which comprises eight subunits (Sec3, Sec5, Sec6, Sec8, Sec10, Sec15, Exo70, and Exo84), mediates the tethering of secretory vesicles to the plasma membrane prior to SNARE-mediated fusion (soluble N-ethylmaleimidesensitive factor attachment protein receptors [67]). Hase et al. proposed that the exocyst complex mediates M-Sec-induced membrane protrusion by promoting two processes: remodeling of the actin cytoskeleton and membrane supplementation [66]. This work was confirmed and completed by Schiller et al. [68]. LST1 (leukocyte-specific transcript 1), a transmembrane protein that is highly expressed in macrophages and dendritic cells, positively affects the number, length, and functionality of TNTs (tunneling nanotubes) in HeLa cells [68]. Since LST1 coimmunoprecipitates and colocalizes with RalA, M-Sec, and Sec5 at the plasma membrane, the authors proposed that LST1 acts as a membrane scaffold that recruits RalA and its effector, filamin, as well as M-Sec and the exocyst complex, to the plasma membrane. Similar conclusions were drawn using 5637 bladder cancer cells [69], in which RalA GEF RalGPS2 interacts with LST1 and is required for the formation of TNTs in these cells, likely through a pathway involving the RalA and Sec5 proteins. M-Sec is also a positive regulator of TNTs in the U2OS osteosarcoma cell line. There, it interacts with nucleolin (NCL), an RNA-binding protein, which would act upstream of 14-3-3ζ to regulate p-cofilin levels [70]. It also interacts with the endoplasmic reticulum chaperone protein ERp29, which could be required upstream M-Sect. [71]. Interestingly, the presence of Erp29 in the TNT proteome [34] suggests a role outside the ER or the transfer of ER fragments through the TNTs, maybe allowing local translation.

Therefore, Rho GTPases (and ARP2/3, which also binds to the exocyst complex) can regulate secretory vesicle movement and tethering to the membrane, as well as actin remodeling (steps 1, 2). These two steps could be necessary for TNT formation.

The cell cortex is typically defined as a thin layer of an actin meshwork underlying the plasma membrane of an entire cell. It is the main determinant of the cell surface’s stiffness and resistance to external mechanical stresses. However, the structure and subcellular distribution of the cortex vary significantly across cell types (in particular adherent vs. cells in suspension) and the cell’s physiological state [72]. Together with the different amounts of Rho GTPases, effectors, and actin regulators in various cell types, this could explain why different results have been obtained in neuronal cells. In CAD neuronal cells, CDC42 behaves as a negative regulator of TNTs when working with VASP (vasodilator-stimulated phosphoprotein) and IRSp53. Overexpression of active forms of these proteins, either individually or collectively, affects the number and functionality of TNTs [73] and provokes an increase in filopodia formation.

Another recent study illustrates the complexity and versatility of TNT formation regulation by Rho GTPases. Rho-associated kinase (Rock/Rok), an effector of the small GTPase Rho, belongs to the AGC family of kinases and can be inhibited by Y-27,632. Using retinal pigment epithelial (RPE) cells, Yuan et al. [74] demonstrated that Y-27,632 increases the number and length of TNTs, as well as mitochondrial transfer, in a dose-dependent manner, confirming and extending previous results in A549 lung adenocarcinoma cells [53]. In these cells, mitochondrial transfer mainly occurs through tubulin-containing TNTs. The inhibitor increased the number of these TNTs, although TNT formation primarily hinges on actin filaments. The negative effect of Rock was also confirmed in other cancer cells [46, 57, 75]. Based on these findings, one could speculate that Rho GTPases may reinforce already formed TNTs by polymerizing microtubules (step 4).

### Small Rab GTPases

Because they have been regarded as one of the central hubs for membrane trafficking, small rab GTPases (comprising 60 gene products [76]) were tested for their participation in TNT regulation. Like other small GTPases, Rabs cycle between two states: an active (GTP-loaded) state and an inactive (GDP-loaded) state. Inactive (GDP-loaded) Rabs are extracted from membranes by GDIs, which mask their prenylated C terminus. Next, Rabs must be activated (GTP-loaded) by GEFs. Once activated, Rabs localize to specific membranes of several compartments, such as the endoplasmic reticulum (ER), the Golgi apparatus, secretory vesicles, endosomes, and lysosomes. There, they recruit effector proteins, i.e., active Rab-binding partners, that regulate different steps of membrane trafficking.

Zhu et al. tested the role of Rab GTPases in TNT formation by overexpressing 41 GFP-Rab GTPases in neuronal CAD cells and using cell-to-cell vesicle transfer as a readout [77]. Of the rabs that affect vesicle transfer, rab8A, already described as positively affecting TNTs in HeLa cells [78] and rab11A (also positively affecting TNT number in human epithelial bronchial or mesothelial cells lines [79]) were studied in more detail. They were both shown to have a positive effect on the number and functionality of TNTs [77]. Epistasis experiments revealed that rab11A acts upstream of rab8A in TNT formation and that the two proteins colocalise at the base of TNTs. Interestingly, the formation of TNTs is dependent on the recycling of the membrane from endosomes, a process involving Rab8 and Rab11. The v-SNARE VAMP3 acts downstream of Rab11 and Rab8 to regulate the formation of TNTs [77], suggesting that this cascade is involved in steps two and three by increasing the recycling of vesicles to the cell surface. However, Ganti et al. recently discovered that Rab11a can move through TNTs in a bidirectional manner alongside influenza A virions in Madin-Darby canine kidney (MDCK) cells without affecting TNT formation [80].

Rab35 was also found to have a positive effect on TNTs [77, 81]. It uses ACAP2/centaurin-β2 as an effector; the latter is, in turn, a GAP (GTPase-activating protein) for ARF6 (ADP-ribosylation factor 6), which negatively regulates TNTs. Following Rab35 activation in the case of neurite outgrowth, ARF6 must be inactivated to maintain PI4P and recruit EHD1 [82]. Similarly, Bhat et al. demonstrated that Rab35 regulates TNTs through ACAP2, ARF6-GDP and EHD1, eventually supplying vesicle membrane for the step 2 of TNT formation. However, MICAL-L1 and MICAL1, two Rab35 effectors that regulate neurite outgrowth [82] and cell abscission following cell division [83], had no impact on the formation of TNTs in CAD cells [81]. This suggests that the regulation of TNTs differs from that of neurite outgrowth and cell abscission, although common regulators are used.

These studies highlight the importance of the precise regulation of the various steps in TNT formation by Rho GTPases, as well as the potential for differences in different cellular contexts. Undoubtedly, future work using cutting-edge tools, such as optogenetic tools [84], will further explore the spatiotemporal dynamics of TNT formation and identify guanine nucleotide exchange factors (GEFs), effectors and GTPases. Additionally, a thorough analysis of the lipids present on the TNT membrane would help to elucidate their origin and the membrane trafficking steps involved in their formation.

## Actin regulators

From six actin genes in mammals, two types of very similar globular actin molecules are translated: four muscle-specific and two non-muscle. These molecules can bind with two other actin monomers via head-to-tail interactions, thus polymerizing into filaments (F-actin). These filaments have distinct polarity; the plus (or barbed) end elongates five to ten times faster than the minus end. Actin monomers bind to ATP, making them more readily available for polymerization. ATP is then hydrolyzed into ADP upon incorporation into the filament. Depending on the equilibrium between actin monomers and filaments, filaments can also depolymerize, resulting in the breakdown of actin filaments. The assembly and disassembly of actin filaments, their crosslinking into bundles and networks, and their association with the cell membrane are regulated by a variety of actin-binding proteins (ABPs). For example, cofilin enhances the dissociation rate of actin monomers bound to ADP from the minus end, thereby disassembling filaments. In contrast, profilin reverses the effect of cofilin by stimulating the exchange of ADP for ATP bound to cofilin-bound actin. This results in the dissociation of actin monomers from cofilin, followed by their assembly into filaments. Other proteins, called nucleators (e.g., ARP2/3 proteins), initiate the assembly of new filaments by stabilizing or mimicking small actin oligomers. This results in accelerated polymerization and the formation of branched actin networks. Capping protein (CP) binds the fast-growing barbed end of the actin filament and regulates actin assembly by blocking the addition and loss of actin subunits, thereby fine-tuning actin assembly dynamics [85]. Their interaction with actin may promote nucleation of new filaments, increase branching of filaments, and/or enhance filament depolymerization. Other factors have bundling activity, which allows for the crosslinking of actin into closely packed, parallel filaments (e.g., α-actinin, villin, and fimbrin) or into orthogonal filaments (e.g., filamin). The physical properties and organization of the resulting actin cytoskeleton differ depending on whether meshwork (semisolid gels) or bundle (more rigid) patterns are favored. Bundles with enough stiffness must be formed to get around buckling and membrane resilience issues, and to enable protrusion growth. Due to the peculiar structure of the TNTs, which are supported by specific actin bundles, actin regulators may be recruited at different stages of TNT formation and in a manner that is distinct from that of other actin-based protrusions.

Treating cell cultures with chemical actin-depolymerizing agents (e.g., latrunculin or cytochalasin D) leads to decreased TNT numbers in several cell models [43, 68, 86–88]. However, jasplakinolide, an actin-polymerizing and -stabilizing drug, also blocked TNT communication between glioblastoma cells and astrocytes [65]. This suggests that proper TNT growth requires actin depolymerization and repolymerization at different time points, as outlined in steps 1 and 3.

Which actin-related proteins are known to play a role in TNT biology? As there is still no clear picture of the sequence of intervention and the potential interactions of these factors, we will present them based on their role in actin polymerization.

### Actin nucleation

The key actin partner for the nucleation of branched actin filaments is the ARP2/3 (Actin Related Protein 2/3) complex, constituted of seven protein subunits (ARP2, ARP3 and ARP complex-1 (ARPC1) through ARPC5). The ARP2/3 makes a new actin branch on a pre-existing “mother” filament. Because of the ~ 70° branching angle, repetitive rounds of ARP2/3-dependent nucleation produce actin filaments oriented at a variety of angles. These filaments are also very short, because their growth is blocked by capping protein soon after nucleation. As a result, branched actin networks are often dense and nearly isotropic and consist of relatively short filaments.

Using the ARP2/3 inhibitor CK666, Hanna et al. [65] observed a decrease in the number of TNTs in macrophages, also described in A549 lung adenocarcinoma cells [53]. Activation of the Rho GTPase effectors WASP or WAVE2 in macrophages induces a conformational change that allows interaction with ARP2/3, thereby activating it. However, in other cell types where cdc42 has an opposite effect on TNTs, CK666 stimulates TNT formation. This has been observed in neuronal CAD cells, where knocking down one of ARP2/3 subunit increased the number of TNTs [30, 42] and increased the transfer of protein aggregates [89]. CK666 has also been used in vivo with opposite results: primary cardiac cells treated for 15 min or hearts of E9.25 embryos whose mothers were treated with CK666 exhibit fewer TNT-like connections [10]. Human trabecular meshwork cells treated with CK666 for 2 h display less and shorter filopodia, and decreased transfer through TNTs [90]. In zebrafish gastrulae, CK666 treatment for one hour resulted in an increased length of TNTs [9], with no effect on the number of connected cells.

CK666 releases the pool of actin from the branched actin network, thereby facilitating linear polymerization and enabling the formation of longer connections. This is consistent with its positive effect on the number of TNTs. However, the ultrastructure of TNTs in different cell types remains unclear. There may be slight differences compared to neuronal cells, which could explain why the ARP2/3 complex (or at least CK666) plays an opposite role. As previously mentioned, the organization of cortical actin could differ, as could the balance between all actin regulators and the propensity of cells to grow TNTs rather than filopodia.

### Actin bundling

Colinear assemblies of uniform polarity of filamentous actin (F-actin), called bundles, are prominent in tubular membrane protrusions such as filopodia and TNTs. In vitro and in filopodia, fascin cross-links actin filaments (F-actin) into hexagonal arrays [91]. This latter arrangement has not been observed in TNTs of neuronal cells [30]. The minimal two-component fascin and F-actin system recapitulates much of the previously described complexity of filopodia cores. However, fascin can collaborate with additional cross-linkers to build other protrusions, and multicross-linker networks may feature distinct architectures and functional properties. Interestingly, like VASP, fascin has been described as both a positive regulator of filopodia formation and a negative regulator of TNT formation in CAD cells [92]. This is in accordance with the different architecture of actin bundles in filopodia versus TNTs. It is not yet known whether the balance between filopodia and TNTs changed in favor of filopodia when fascin was overexpressed, leaving less G-actin available to incorporate into growing TNTs; whether the fascin effect was active on TNTs; or whether fascin requires additional cross-linkers to build TNT bundles. In general, the overexpression or inhibition of actin regulators can affect several aspects of cells; the resulting effect on TNTs may be a side effect.

Filamin (FLN) proteins are a family of three proteins (FLNA, FLNB, and FLNC) that are products of distinct genes. They are known to serve as scaffolds for over 90 binding partners, including integrin beta, Rho GTPases, GEFs and GAPs of rho GTPases, as well as transcription factors [93]. FLN consists of two large subunits that self-associate to form a long, semi-flexible strand. Each subunit has an N-terminal actin-binding domain, followed by β-strand repeats that can form a binding pocket for an opposing β-strand donated by a binding partner. These interactions are regulated by mechanical forces, FLN phosphorylation, and proteolysis, as well as competition between binding partners and/or multimerization of partners. FLN isoforms have both common and distinctive binding partners, as well as features in their structures, expression levels, and localizations. Mass spectrometry analysis of co-precipitated material identified filamin, myoferlin and the myosin II heavy chains MYH9/MYH10 interacting with overexpressed LST1 in Hela cells. Based on these interactions and on colocalization to the plasma membrane, LST1 was proposed to recruit the small GTPase RalA and its effector filamin to the plasma membrane, thereby inducing localized actin-crosslinking (step 3) [68]. Note also that the 3 filamin proteins were identified in the mass spectrometry analysis of TNT fractions from U2OS cells [34]. However, no experiment has yet been performed that directly assesses the role of filamin in TNT formation.

Epidermal growth factor receptor kinase substrate 8 (Eps8) is a signaling adapter that controls various cellular protrusions by regulating the dynamics of the actin cytoskeleton. Initially, it was described as controlling actin-based motility by capping the barbed ends of actin filaments when associated with ABI1 [94]. Eps8 also exhibits actin bundling activity, which enhances membrane extensions and promotes filopodial protrusions [94]. When overexpressed in neuronal cells or in zebrafish gastrulae, Eps8 positively regulates TNT formation and function through its bundling activity [42, 73]. Its capping activity is not required and may even be inhibitory [9]. Interestingly, Eps8 forms a complex with IRSp53 (insulin receptor tyrosine kinase substrate of 53kD, also known as BAIAP2), which binds to the membrane and both cooperate in bundling actin filaments for filopodia formation in Hela cells [94]. See later sections for more details about IRSp53 participation in TNT formation.

### Actin capping

F-actin capping proteins, such as actin capping protein (CP), gelsolin, villin, severin, fragmin, adseverin, and scinderin, bind to the rapidly growing barbed end of F-actin filaments [95]. They regulate actin assembly by blocking filament growth and/or depolymerization. Most of these proteins have severing activity in addition to capping activity. Capping proteins can interact with regulators that modulate their activity. They can also be indirectly regulated by proteins that compete with them for binding to the barbed end of actin. This is the case for ENA/VASP (Enabled (ENA), vasodilator-stimulated phosphoprotein (VASP)) proteins [96]. ENA/VASP proteins accelerate filament elongation by delivering actin monomers to the growing barbed end and contribute differently to lamellipodia protrusion versus microspike and filopodia formation [96]. Interestingly, VASP overexpression decreased CAD cell ability to form TNTs and concomitantly increased dorsal filopodia [73, 92]. VASP and the two subunits of capping protein, CAPZA1 and CAPZB, which do not sever actin, were found in TNT proteome analysis, but no other capping proteins were found [34].

### Actin depolymerization

Cofilin, a small actin-binding protein belonging to the actin depolymerizing factor (ADF)/cofilin family, promotes F-actin disassembly. Cofilin 1, the non-muscle isoform, has been shown to play pivotal role in the function and plasticity of dendritic spines [97], in outgrowth of axons [98] and in ciliogenesis [99], always by participating in the dynamic restructuring of actin cytoskeleton.

Cofilin binds both globular (G)-actin and filamentous (F)-actin but with a strong preference (> 40 fold) for binding ADP-actin. At low concentrations with respect to actin subunits (1:750), cofilin is an effective F-actin severing protein. At higher concentrations, severing efficiency declines, but this occurs at the junction between cofilin-saturated and unsaturated regions. This shows that the local quantity and activity of cofilin matter. However, stable cofilin-actin complexes can also nucleate growth [100]. Cells utilize many mechanisms to locally control cofilin, including post translational modifications (phosphorylation, ubiquitinylation, neddylation, Oxidation-Reduction), binding to phosphatidylinositol phosphates (PI-4P and PI-4,5P2; PIPs) and cooperation/competition with other proteins. Phosphorylation deactivates cofilin and releases it from actin. This inhibits cofilin’s ability to sever and depolymerize F-actin. Consequently, the cellular concentration of G-actin decreases, as does the turnover rate of actin filaments. The phosphorylation of Cofilin is regulated by LIMK1 (LIM kinase 1), a membrane-anchored protein, as well as by other proteins, such as TESK1 and 2 and protein kinase Cα (PKCα). Although LIMK activation mostly results from Rho, Rac, or Cdc42 signals, many other pathways can lead to cofilin phosphorylation [100]. No study has addressed the specific contributions of each pathway to TNT regulation, and only two articles involve cofilin in TNT regulation. In MCF7 cancer cells, oxidative stress increases the number of TNTs in a cofilin-dependent manner. Oxidative stress decreases phospho-cofilin species. Inducing cofilin dephosphorylation with the ROCK-specific inhibitor Y-27,632 leads to the same result, indicating that cofilin is a positive regulator of TNTs [46]. Similarly, a previous study by Dagar et al. [70] showed that depleting M-Sec, NCL, and 14-3-3 zeta one by one is correlated with decreased phospho-cofilin and increased TNT numbers in mammalian osteosarcoma (U2OS) cells. 14-3-3 zeta would act downstream of NCL, and M-Sec would interact directly with NCL, but also act by another way on cofilin phosphorylation [70].

The data on the steps and pathways regulated by M-Sec show how difficult it is to draw universal conclusions from data obtained in various cell lines. Additionally, most studies did not address which step different factors, including cofilin, regulate. Given its potential role in severing actin and its positive impact on TNT quantity, we hypothesize that cofilin influences the initial stage (step 1) of TNT formation.

The MICAL (microtubule-associated, monooxygenase, calponin and LIM domain-containing) protein family is encoded by three genes in the human genome: MICAL1, MICAL2 and MICAL3. A group of MICAL-like proteins has also been identified, including MICAL-L1 and MICAL-L2, which lack the N-terminal region involved in actin oxidation and disassembly [101]. Members of the MICAL family are involved in various cellular processes, such as cell division, migration, and endo- and exocytosis. These functions involve F-actin depolymerization [83]. MICAL1 and MICAL3 harbor Rab binding sites, making them Rab effectors, however MICAL-L1 and MICAL1 were shown not to affect the formation of TNTs in CAD cells [81]. Two splicing variants of MICAL2, MICAL2PVa and MICAL2PVb, which are detectable in prostate and lung cancers, negatively affect TNT number and functionality [102] by regulating the actin cytoskeleton. Consequently, mitochondrial distribution and trafficking are affected, resulting in modulated responses of lung cancer cells to chemotherapeutic drugs, likely by suppressing their resistance [102]. However, it is not known whether MICAL2PVs impair TNT growth (step 3) or enhance their degradation (step 7), and the ability of MICAL2 to perform the same function as a negative regulator of TNT has not yet been assessed.

### Stabilization of actin filament

Ca²⁺/CaM-dependent protein kinase II (CaMKII) belongs to a family of multifunctional CaMKs. The four members of the CaMKII subfamily—α, β, γ, and δ—are differentially expressed in various tissues and exhibit different levels of actin-binding activity. The β isoform is particularly adept at binding to F-actin. At low Ca²⁺ concentrations, CaMKIIβ binds to actin filaments, bundling them together and preventing depolymerization. CaMKIIβ also binds to G-actin, thus preventing nucleation [103]. Raising the Ca²⁺ concentration activates another signaling protein, calmodulin. Calmodulin then triggers the dissociation of CaMKII from F-actin bundles by binding to CaMKII. This dissociation creates a time window in which F-actin can be modified by actin-regulatory proteins. Additionally, CaMKII can directly interact with microtubules and other actin-regulatory molecules, including IRSp53 [103, 104]. CaMKII also regulates the small GTPases cdc42, rhoA, and rac1, which are upstream of actin remodeling events, notably in TNT formation. Using different CaMKIIβ mutants and a pharmacological inhibitor, Vargas et al. demonstrated that the actin-binding activity of CaMKIIβ is necessary to increase the number of TNT-connected neuronal CAD cells [36]. This effect could be due to an increase in the de novo formation of TNTs or an increase in the stabilization of already formed TNTs. TNTs have a longer half-life when the actin-binding activity of CaMKII β is not impaired; however, this does not depend on its kinase activity [36]. Upon a calcium signal (e.g., activation of the Wnt signaling pathway), CaMKII phosphorylation could detach from F-actin, allowing access to actin-regulator proteins (step 1). Additionally, after actin polymerization and initiation of TNT formation, CaMKIIβ could become dephosphorylated and reattach to F-actin, thereby increasing the stability of the formed TNTs (step 7).

## Actin and membrane bond proteins

The formation of TNTs requires rearranging the three-dimensional network beneath the plasma membrane. This network consists of actin filaments and adhesion-binding proteins (ABPs), which are organized as the cell cortex. Depending on the cell type, the cell cortex is associated with the plasma membrane through various means, either along the entire membrane or at specialized regions such as focal adhesions or adherens junctions. Therefore, proteins that link the cell membrane to the F-actin network are key to remodeling this microenvironment and pushing out the new protrusion (steps 1, 2, and 3).

IRSp53 is an adapter protein that links membrane-bound small G-proteins, particularly activated Rac1 or Cdc42, to cytoplasmic effector proteins. IRSp53 also contains a crescent-shaped inverse BAR (Bin/Amphiphysin/Rvs) domain that can induce, sense, and stabilize the negative curvatures found in cellular protrusions, such as filopodia and TNTs [105]. IRSp53 also has an SH3 domain that recruits regulators of actin polymerization, such as vasodilator-stimulated phosphoprotein (VASP), Mena, Eps8, mDia1, and WASP-family verprolin-homologous protein 2 (WAVE2) [105]. Due to these characteristics, IRSp53 constitutes a functional platform at the interface between the plasma membrane and the actin cytoskeleton. It mediates Cdc42-dependent, actin-based events [94]. Eps8 can positively or negatively affect filopodia formation, depending on the context (positive in HeLa cells and negative in primary hippocampal neurons). A second IRSp53 interactor, VASP, competes with Eps8 and can cause filopodia formation independently [106]. Both IRSp53 and VASP were initially described as negative regulators of TNTs when overexpressed in neuronal cells [73]. However, more recent work using overexpression (OE) and knockdown (KD) approaches revealed IRSp53 to be a positive regulator of TNT number and function in CAD cells, acting cooperatively with Eps8 [42]. Therefore, IRSp53 can have a versatile role, depending on its association with VASP, eps8, or others. The formation of actin filament bundles has been proposed as necessary for efficient protrusion by filling the space and providing mechanical support for initial membrane deformation during filopodia extension [107]. IRSp53 and its cofactors could induce this initial bundling in TNTs (step 2). However, Eps8 and IRSp53 co-overexpression also increases TNT duration, suggesting an additional role in TNT stability (step 7). In C2C12 myoblasts, CUL3KCTD10, an E3 ubiquitin ligase, is necessary for cell-to-cell fusion by targeting Eps8 [108]. Eps8-IRSP53 complexes stabilize cortical actin bundles at sites of cell contact and push the membrane outward to promote close membrane alignment. When Eps8 or IRSp53 is reduced, fusion occurs in the absence of CUL3KCTD10. Eps8 monoubiquitinylation by CUL3KCTD10 displaces the complex from cell-contact sites, counteracting actin bundling and allowing progression toward cell fusion. In the absence of CUL3KCTD10, however, the complex accumulates at cell-cell contact sites, impairing fusion. Based on these data, one could also hypothesize that Eps8/IRSP53 is involved in steps 3 and 5 (protrusion growth and TNT fusion with the target cell). However, no screen has been performed yet to identify factors that trigger the disassembly of actin-regulating complexes in the context of TNTs.

Myosins are actin-based motor proteins that have ATPase activity. Myosin 10 (Myo10) is an unconventional motor protein that localizes to cell protrusions, including filopodial tips [109]. Myo10 binds to actin filaments and bundles, functioning as a plus-end-directed motor that moves with higher velocity and takes larger steps on bundles than on single filaments [110]. In addition to its motor domain, Myo10 can interact with membranes containing phosphatidylinositol-3,4,5-trisphosphate via its PH domains. This membrane binding opens its conformation, increasing its actin-dependent ATPase and motor activities. Myo10 can also interact with tubulin, integrins, cadherins, calmodulin, and, of course, its different cargoes. In neuronal cells and macrophages, as well as likely in human trabecular meshwork cells, Myo10 positively affects the number and function of TNTs [92, 111, 112]. This function requires the full-length protein, except for a portion of the FERM domain, which is involved in the interaction with integrins and integrin-dependent elongation of filopodia [113]. Myo10 could facilitate the movement of cargo on actin bundles and/or microtubules within TNTs (step 6). However, its effect on increasing the number of TNTs may be indirect since it can interact with Eps8 [42] or CamKIIa/b [104].

In addition to Myo10, our recent proteomic analysis [34] identified several other motor proteins associated with TNTs from U2OS cells, including members of the myosin family (Myosin1C, MYL12A, MYH9), tropomyosins (Tropomyosin 1, 2, 3 and 4), dyneins, dynactins, and kinesin (KIF5B). On the other hand, it was shown that inhibition of Myo1d could reduce the alpha-synuclein PFF transmission from BMVECs to pericytes [114], suggesting that various motor proteins can be required for TNT-dependent propagation, depending on the cell type, cargo and ultrastructure. They could affect the transfer through TNTs without participating directly in the formation of TNTs.

Several annexins (ANXA2, 5, 1, 6, 4, 1, 7, and 3) were among the most abundant proteins identified in the TNT proteome [34]. These proteins are of high interest because they bind to calcium, lipids, and F-actin. They have been shown to mediate the formation of membrane domains that support docking and fusion events, as well as the repair of damaged membranes and the stabilization of cortical actin [115]. Our preliminary work identified ANXA2 as a negative regulator of TNT (personal communication).

## Transmembrane proteins

Connexin 43 (Cx43), which is encoded by the GJA1 gene, is the most widely expressed and studied connexin. It is a transmembrane protein that forms hexameric pores, or hemichannels, in the cell membrane. These hemichannels can communicate with the extracellular space and/or dock with channels on opposing cells to form gap junctions. This process affects direct intercellular communication. Some TNTs that are positive for Cx43 at one end of the connection have been shown to allow electrical coupling through interposed gap-junction channels [116, 117]. Sun and Zhao further demonstrated using C2C12, HEK, and A549 cells that Ca²⁺ influx and Cx43 play roles in TNT-mediated electrical coupling induced by mechanical stimulation [118]. The proportion of TNT-mediated electrical coupling decreased significantly in C2C12 cells when treated with a drug that inhibits gap junctions (MFA). The localized accumulation of Cx43 at the TNT ends once again suggested its potential involvement in forming electrical coupling sites between connected cells.

In addition to its role in forming gap junctions, Cx43 has channel-independent functions. These include the modulation of mitochondrial dynamics [119] and the regulation of lysosome exocytosis via ARP2-mediated remodeling of the actin cytoskeleton [120]. The loss of Cx43 expression has been associated with a significant reduction in TNT length and number in breast cancer cell lines, in which Cx43 could directly regulate intracellular signaling pathways that affect TNTs [57]. Furthermore, Cx43 undergoes alternative translation to generate various N-terminally truncated isoforms, notably GJA1-20k. GJA1-20k aids in mitochondrial motility by mobilizing mitochondria along microtubules [121]. It also increases mitochondrial transfer from astrocytes to neurons [122], from transplanted mesenchymal stem cells (MSCs) derived from induced pluripotent stem cells (iPSCs) to airway epithelial cells [123], and from human mesenchymal stromal cells to chondrocytes [20, 124]. The latter case is particularly interesting since mitochondrial dysfunction is one of the earliest cellular responses to traumatic injury in cartilage tissue, which can lead to osteoarthritis. Cartilage tissue consists of chondrocytes embedded within a dense extracellular matrix (ECM). It has limited self-repair capacity, partly due to its avascular nature. Oxidative stress increases the transfer of mitochondria from mesenchymal stromal cells (MSCs) to chondrocytes, primarily via tunneling nanotubes (TNTs), which have increased connexin 43 (Cx43) staining. Decreasing Cx43 in MSCs reduces mitochondrial transfer to chondrocytes. Conversely, GJA1-20k promotes the transport of mitochondria from MSCs to neighboring cells. Cx43 and GJA1-20k may increase mitochondrial transfer by promoting the mobilization of mitochondria through TNTs, by encouraging microtubule polymerization within them [125], or by participating in F-actin remodeling [126] (steps 1–6). It may also affect the secretome to induce TNT formation [57] or the trafficking of full-length Cx43 hemichannels to the cell membrane. Therefore, TNTs could have two complementary functions: electrical coupling with connected cells through Cx43-containing gap junctions and the transfer of large cargo partially through Cx43’s channel-independent function. Accordingly, blocking or reducing gap junction (GJ) communication during HIV infection results in aberrant tunneling nanotube (TNT) cell-to-cell contact, which compromises HIV spread and replication [48].

N-cadherin has been observed along TNTs and at the connection point between TNTs and target cells in various cell models, including urothelial cell lines [127], neuronal cells [30, 44], and HeLa cells [128]. In the latter case, the authors describe how close-ended protrusions form through the twisting of a double filopodial bridge (DFB). This process may not result in true TNTs. Nevertheless, these connections, through which Ca²⁺ ions can be transferred unidirectionally, are tightly linked to the paired cell body via N-cadherin interactions. Their formation is N-cadherin dependent [128]. A recent functional study on N-cadherin in neuronal cells has shown that N-cadherin acts as a positive regulator of TNTs through two complementary mechanisms: cross-linking individual TNTs (iTNTs) into parallel bundles that provide structural support (step3) and reinforcing TNT tip adhesion to recipient cells to facilitate docking and fusion (step 5). N-cadherin works via its effectors, α-catenin and p120-catenin, to ultimately affect the Cdc42–IRSp53–N-WASP pathway [44]. N-cadherin was found in the TNT-enriched fraction of UOS cells [34], supporting its conserved role in non-neuronal cells.

The tetraspanin family comprises 33 similar-structured members in mammals. These small proteins have four membrane-spanning domains and two extracellular domains, including one large domain with a tetraspanin-specific fold and two short cytoplasmic tails. Tetraspanins regulate the trafficking and subcellular localization of partner proteins, including other transmembrane proteins. Together, they form a dynamic network of molecular interactions. CD9 and CD81, in particular, are primarily expressed at the plasma membrane and are commonly used as extracellular vesicle (EV) markers. They also decorate ectosomes, midbody remnants, and migrasomes [129]. However, their specific function remains elusive. For example, their role in EV production, EV attachment, and EV fusion with target cells is controversial [129].

A mass spectrometry analysis of the TNT-enriched fraction in U2OS cells revealed that CD9 and CD81 are among the most abundant integral membrane proteins in TNT and that their direct interacting partners, CD9P1 and EWI2, are also present [34]. A common feature of cell structures that are positive for CD9 and CD81 is their high curvature. Thanks to the cone-shaped structure of the tetraspanin intramembrane domain, tetraspanins can sense and influence the shape of membrane structures. Our data on SH-SY5Y cells show that CD9 and CD81 are positive regulators of TNTs. They work partially redundantly and partially through different mechanisms. CD9 positively regulates the number of TNT-connected cells and vesicle transfer, likely by stabilizing TNTs. Treatment with a CD9 antibody also relocates CD9 and CD81 to TNT extremities. This supports the idea that CD9-containing complexes play a role in stabilizing TNTs, perhaps by favoring cis and/or trans interactions, particularly between iTNTs (steps 2 and 3). In contrast, CD81 regulates vesicle transfer without affecting the apparent number of connected cells. Nevertheless the number of fully completed TNTs is lower in the absence of CD81 [34]. CD81 could therefore participate in anchoring opposite membranes and, finally, opening the channel (step 5). Notably, CD9, CD81, and their partners impact cell-to-cell fusion in other contexts [130–132]. Whether this is also the case during TNT formation remains to be formally proven. Since N-cadherin and tetraspanins affect the same step in TNT formation, it would be also interesting to address the following questions: Does CD9/CD81 interact with N-cadherin or other transmembrane proteins in specific complexes on TNTs/iTNTs? Do N-cadherin and tetraspanins act independently, sequentially, or cooperatively?

##  Others

In human epithelial bronchial or mesothelial cell lines, depleting the RASSF1 (Ras association domain family member 1) tumor suppressor gene increased the number and length of functional TNTs. RASSF1 influences various pathways that independently affect TNT number and functionality: vimentin expression, LIMK/cofilin activity, exosome release, GEF-H1 activity (a GEF of RhoB GTPase), and Rab11 accumulation [79, 133]. However, RASSF1A also plays roles in regulating apoptosis, the cell cycle, genome integrity, and tumor cell adhesion and motility. This illustrates once more how difficult it is to decipher TNT regulation alone.

## Conclusions

The formation of TNT relies on versatile mechanisms, and current knowledge is insufficient to provide a comprehensive understanding of how external or internal stress signals prompt cells to grow these specific structures at specific (or non-specific) subcellular locations and towards specific (or non-specific) target cells. Yet other key questions remain: how TNTs are distinguished from other protrusions, how cargo selection and directionality are regulated, and how their activity interfaces with extracellular vesicles and gap junctions. Addressing these challenges will require high-resolution imaging, single-cell transcriptomics, and innovative perturbation strategies. A deeper mechanistic understanding may not only unify current models of TNT biogenesis but also identify actionable targets for modulating TNTs in cancer, infection, and neurodegeneration.

## Data Availability

Not applicable.
